# Associations of serum folate and vitamin C levels with metabolic dysfunction-associated fatty liver disease in US adults: A nationwide cross-sectional study

**DOI:** 10.3389/fpubh.2022.1022928

**Published:** 2022-10-26

**Authors:** Yuqi Jiang, Huanyi Cao, Xingying Chen, Genfeng Yu, Cheng Song, Hualin Duan, Feng Tian, Heng Wan, Jie Shen

**Affiliations:** ^1^Department of Endocrinology and Metabolism, Shunde Hospital, Southern Medical University (The First People's Hospital of Shunde Foshan), Foshan, China; ^2^Department of Endocrinology, Guangdong Provincial People's Hospital, Guangdong Academy of Medical Sciences, Guangzhou, China; ^3^Health Management Division, Shunde Hospital, Southern Medical University (The First People's Hospital of Shunde), Foshan, China; ^4^Department of Endocrinology, The Third Affiliated Hospital of Southern Medical University, Guangzhou, China

**Keywords:** folate, vitamin C, metabolic dysfunction-associated fatty liver disease, vibration-controlled transient elastography, National Health and Nutrition Examination Survey

## Abstract

**Background:**

Clinical research results on the relationship between folate and non-alcoholic fatty liver disease are contradictory. Metabolic dysfunction-associated fatty liver disease (MAFLD) is a recently proposed concept. Evidence about the relationship between serum folate and MAFLD, especially considering the status of serum vitamin C, is scarce. This study was aimed to investigate the association of serum folate levels with the prevalence of MAFLD, and further to analyze the potential impact of serum vitamin C status on their association.

**Methods:**

Totally 2,797 participants from National Health and Nutrition Examination Survey (NHANES) 2017–2018 were included. Vibration-controlled transient elastography was used to detect liver steatosis and fibrosis. Participants were divided in groups based on the tertiles of serum folate or vitamin C, and the serum folate or vitamin C level in T1 was low. Logistic regression analysis in the complex sample module was performed to illustrate the association of serum folate levels with the prevalence of MAFLD. Stratification analysis by serum vitamin C status was performed as well.

**Results:**

Compared with the serum folate levels of T1 group, participants in the T3 group had 47.9% lower risk of MAFLD [OR = 0.521 (95% CI: 0.401–0.677)]. However, when participants were stratified by serum vitamin C levels, there was no association between the serum folate levels and MAFLD in the T1 or T2 group. Among participants in the T3 group of vitamin C status, participants in the T3 group of serum folate had a 63.6% lower risk of MAFLD compared with the T1 group [OR = 0.364 (95% CI: 0.147–0.903)].

**Conclusions:**

High serum folate level is associated with lower prevalence of MAFLD, especially in participants with sufficient vitamin C.

## Introduction

Non-alcoholic fatty liver disease (NAFLD) is a very common chronic liver disease with a global prevalence about 25% (1), and affects over 30% of people in the United States (2). In addition to the gradual progression into fibrosis, cirrhosis and hepatocellular carcinoma, NAFLD is also associated with major adverse cardiovascular events and increased all-cause mortality (3–5). Based on the new understanding of fatty liver disease, some international experts recently proposed to replace the term NALFD with metabolic dysfunction-associated fatty liver disease (MAFLD) (6, 7). MAFLD is an inclusive disease that emphasizes the relationship between fatty liver and metabolic status. The definition of MAFLD helps better identify and manage patients at risk of cardiovascular complications, and is more clinically valuable than the definition of NAFLD (8, 9). Therefore, it is crucial to identify the possible risk factors of MAFLD.

Folate is a water-soluble B-complex family of vitamins. The word “folate” includes polyglutamates that are naturally found in foods and folic acids, and is a synthetic form taken as a supplement (10, 11). Folate plays an important role in hepatic DNA methylation, oxidative stress and fat metabolism, and has a major impact on the pathogenesis of NAFLD (12–14). However, the clinical research results on the relationship between folate and NAFLD are contradictory. A small investigation concludes that severe NAFLD is associated with lower serum folate concentration in obese individuals (15). Nevertheless, a study with 30 NAFLD patients and 24 healthy controls reveals no significant inter-group difference in folate levels and no association between folate levels and the severity of liver disease (16). However, the numbers of people observed in these epidemiological studies are relatively small. Furthermore, MAFLD is a recently proposed concept, and its relationship with serum folate has not been reported. Thus, it is essential to clarify the association between serum folate and MAFLD.

Vitamin C, a powerful antioxidant, can scavenge the free radicals produced by the body, and may be related to the pathogenesis of MAFLD (17, 18). The association between serum Vitamin C levels and the prevalence of MAFLD in populations was also demonstrated recently (19). Therefore, it can be reasonably hypothesized that vitamin C status may protect against MAFLD from low serum folate. The use of antioxidant combination therapy may have a positive effect on the improvement of MAFLD and liver fibrosis (20). Furthermore, more than 50% of elders in US took a multivitamin supplement containing both folate and vitamin C, aiming to improve or maintain health (21). Hence, it is necessary to investigate the impact of vitamin C status on the association between folate levels and MAFLD.

Therefore, this study was aimed to examine the association of serum folate levels with the prevalence of MAFLD, and further analyze the effect of serum vitamin C status on the association between serum folate and MAFLD. We present the following article in accordance with the STROBE reporting checklist.

## Methods

### Data source and study population

**Figure 1:**
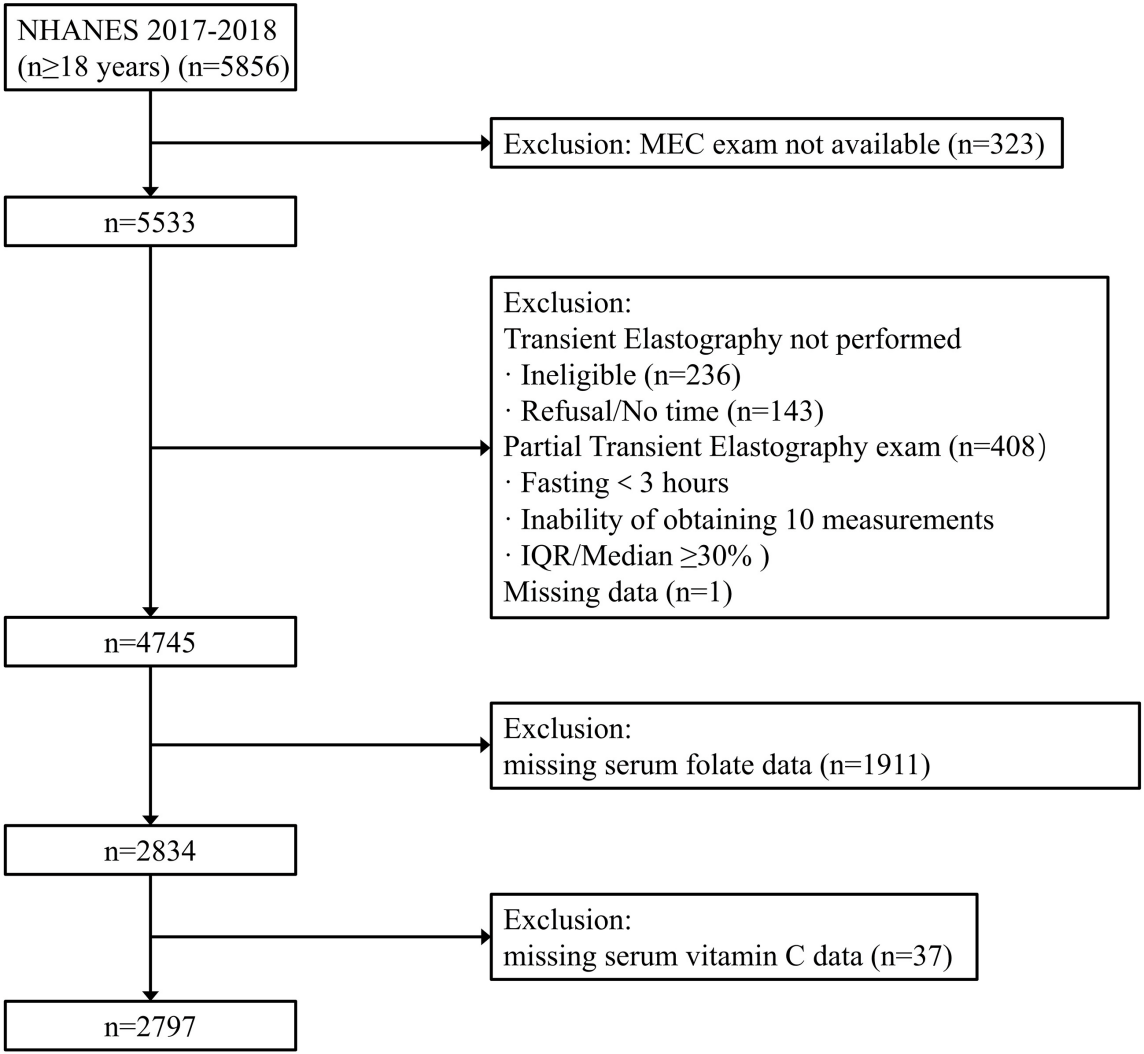
Flow-chart of the MAFLD participants.

The National Health and Nutrition Examination Survey (NHANES) 2017–2018 was adopted, which is a national survey conducted by the National Center for Health Statistics (NCHS) in the United States. To represent the civilian and non-institutionalized U.S. population, NHANES used a stratified, multistage, and clustered probability sampling design. Oversampling was also conducted in certain population subgroups to improve the reliability and accuracy of indicator estimates for these subgroups. This survey met the requirements of the NCHS institutional review board, and all participants signed written informed consent. Our analysis population consisted of 5,856 participants aged ≥18 years from the 2017 to 2018 survey cycle. Participants without Mobile Examination Center (MEC) exam were excluded (*n* = 323). Then 787 participants were excluded because vibration-controlled transient elastography (VCTE) was not performed or their VCTE data was incomplete. We also excluded one person who missed the median controlled attenuation parameter (CAP) scores. Finally, we excluded participants with missing data in serum folate level (*n* = 1,911) or serum vitamin C (*n* = 37), resulting in 2,797 participants in the final analysis (Figure 1).

### Measurement of folate and vitamin C levels in serum

The staff collected whole blood samples and stored them under appropriate conditions. The specimens were shipped to the laboratory and analyzed by trained phlebotomists according to standard protocols. The NHANES quality assurance and quality control (QA/QC) protocols were mandated by the 1,988 Clinical Laboratory Improvement Act.

Serum folate levels were measured using isotope-dilution high performance liquid chromatography coupled to tandem mass spectrometry (LC-MS/MS). Detection was performed by combining each specimen (150 μL of serum) with an ammonium formate buffer and an internal standard mixture. Quantitation was based on peak area ratios interpolated against a five-point aqueous linear calibration curve using 1/*x*^2^ weighting.

Isocratic ultra-high performance liquid chromatography (UPLC) was used to determine vitamin C levels. The peak area of the unknown amount in serum specimens was compared with that of the known amount in the calibration solution to determine the quantitative data of vitamin C.

### Clinical and laboratory data

In household interviews, information such as age, gender, and daily alcohol consumption was collected. Races were classified as Mexican American, Other Hispanic, Non-Hispanic White, Non-Hispanic Black, Non-Hispanic Asian, and others. Education levels were divided into less than high school, high school, and above high school. Smoking status was divided into current, ever, and never. During physical examinations in a mobile examination center, waist circumference and body mass index (weight in kilograms divided by height in meters squared) were collected. Diabetes was defined as a fasting blood glucose level ≥ 7.0 mmol/L, HbA1c ≥6.5%, or a prior diagnosis of diabetes. Hypertension was determined as systolic blood pressure ≥ 140 mmHg, diastolic blood pressure ≥ 90 mmHg, or self-reported hypertension diagnosis. The laboratory methods for measuring total cholesterol (TC) (mg/dL), triglyceride (TG) (mg/dL), high-density lipoproteins cholesterol (HDL-C), and low-density lipoproteins cholesterol (LDL-C) are available in NHANES.

### Definition of variables

Compared to the NHANES from other years, participants in 2017–2018 underwent VCTE examination. VCTE was performed using the FibroScan model 502 V2 Touch (Echosens, Paris, France) equipped with medium (M) or extra-large (XL) probes. Medium probes were used first unless the manufacturer instructions recommended to use XL probes. The detailed procedure manual was described in the Liver Ultrasound Transient Elastography Procedures Manual. Liver steatosis and fibrosis were determined by using the controlled attenuation parameter (CAP) and liver stiffness measurements (LSM) from the VCTE assessment, respectively.

MAFLD was defined on the basis of hepatic steatosis evidence in addition to one of the following three criteria: overweight/obesity, presence of type 2 diabetes mellitus, or evidence of metabolic dysregulation (6). Metabolic dysregulation was defined as the presence of at least two risky metabolic abnormalities: (1) waist circumference ≥ 102 cm in men and ≥ 88 cm women, (2) blood pressure ≥ 130/85 mmHg or specific drug treatment, (3) plasma triglycerides ≥ 1.70 mmol/l or specific drug treatment, (4) plasma HDL-cholesterol < 1.0 mmol/L for men and < 1.3 mmol/L for women or specific drug treatment, (5) prediabetes (i.e., fasting glucose level 5.6 to 6.9 mmol/L, or 2-h post-load glucose level 7.8 to 11.0 mmol or HbA1c 5.7% to 6.4%), (6) homeostasis model assessment (HOMA)-insulin resistance score ≥ 2.5, and (7) plasma high-sensitivity C-reactive protein (hs-CRP) level > 2 mg/L. Liver steatosis was defined as the CAP score ≥ 248 dB/m or ≥ 274 dB/m (22, 23).

### Statistical analysis

IBM SPSS statistical 25 and R 4.1.0 were used for all analyses. According to the NHANES recommendations, appropriate weighting was used for all data analysis. Continuous variables were expressed as mean ± standard error, and categorical variables as frequency (percentage). Differences between continuous and categorical variables were assessed using weighted Student's *t*-test and weighted chi-squared test. A two-tailed *P* < 0.05 was considered significant. Serum folate level was divided into tertiles (24), of which tertile 1 (T1) was used as the reference. Logistic regression analysis in the complex sample module was performed to determine the associations of serum folate levels with the prevalence of MAFLD/NAFLD and liver fibrosis. Logistic regression was also utilized to assess the associations between folate and MAFLD/NAFLD and liver fibrosis stratified by serum vitamin C status. Serum vitamin C levels were also categorized by tertiles (25). The vitamin C levels in T1, T2, and T3 groups were ≤ 39.5 μmol/L, 39.5–61.9 μmol/L, and >61.9 μmol/L, respectively. Model 1 was adjusted for age, sex and race. Model 2 was further adjusted for education, smoking status, and daily alcohol consumption. Stratified analysis was conducted by age and gender to further analyze the association between MAFLD and serum folate, vitamin C. In addition, the associations between serum folate and NAFLD were analyzed. NAFLD was defined as hepatic steatosis excluding viral hepatitis, significant alcohol consumption or the use of steatogenic medications (2). Significant alcohol consumption was defined as >30 g/day for men and >20 g/day for women (26). Liver steatosis was also defined as the CAP scores ≥ 248 dB/m or ≥ 274 dB/m (22, 23). The study population of NAFLD was shown in Supplementary Figure 1. Liver fibrosis was defined as the LSM score ≥ 7 KPa or ≥ 8 KPa (27, 28).

## Results

### Characteristics of participants

Baseline characteristics were compared based on MAFLD (Table 1). A total of 2,797 participants were involved, including 1,318 non-MAFLD participants and 1479 MAFLD participants. Compared with the non-MAFLD group, the MAFLD group tended to be men, older and smokers, and had lower vitamin C serum levels and folate levels.

**Table 1:** Basic characteristics of participants according to MAFLD.

	**MAFLD**		
**Characteristics**	**No (*N* = 1,318)**	**Yes (*N* = 1,479)**	***P***
Age, y	39.07 ± 1.10	48.1 ± 1.25	< 0.01
Sex, %	
Male	37.5	42.9	< 0.01
Female	62.5	57.1	
Race, %	
Mexican American	7.8	13	< 0.01
Other Hispanic	7.3	5	
Non-Hispanic White	61.9	61.8	
Non-Hispanic Black	12.4	9.9	
Non-Hispanic Asian	5.1	5.5	
Other Race	5.5	4.9	
Education, %	
< High school	11.6	11.7	0.58
High school	27.8	29.8	
>High school	60.6	58.5	
Alcohol consumption, g/day	7.12 ± 0.89	6.23 ± 0.87	0.34
Smoking, %	
No	64	58.0	< 0.01
Ever	21.1	26.3	
Current	15	15.7	
BMI, kg/m^2^	25.70 ± 0.37	32.91 ± 0.46	< 0.01
Waist Circumference, cm	88.89 ± 0.94	108.47 ± 1.03	< 0.01
TC, mg/dL	180.83 ± 2.56	188.45 ± 4.46	< 0.01
TG, mg/dL	80.06 ± 2.59	129.57 ± 6.53	< 0.01
LDL-C, mg/dL	105.53 ± 2.17	112.38 ± 3.10	0.07
HDL-C, mg/dL	59.32 ± 1.05	50.46 ± 0.68	< 0.01
Diabetes, %	2.5	24.0	< 0.01
Hypertension, %	14.7	42.5	< 0.01
Vitamin C, μmol/L	52.24 ±1.91	45.82 ± 2.23	< 0.01
Serum folate, nmol/L	39.84 ±1.43	38.08 ± 2.02	0.38

Data were summarized as mean ± SE for continuous variables or as a numerical proportion for categorical variables.

TC, total cholesterol; TG, triglyceride; HDL-C, high-density lipoproteins cholesterol; LDL-C, low-density lipoproteins cholesterol; BMI, body mass index.

### Associations between serum folate levels and MAFLD

Table 2 showed the associations of serum folate with MAFLD. When liver steatosis was defined as CAP score ≥248 dB/m, univariate regression analysis showed that in terms of serum folate, the T3 group had a 27.4% lower risk of MAFLD compared with the T1 group [OR = 0.726 (95% CI: 0.576–0.915)]. After adjusting for sex, age and race, the prevalence of MAFLD in the T3 group decreased by 49.2% compared with the T1 group [OR = 0.508 (95% CI: 0.394, 0.654)]. When we further adjusted for education, smoking status, and daily alcohol consumption, the T3 group of serum folate had a 47.9% lower risk of MAFLD compared with the T1 group [OR = 0.521 (95% CI: 0.401–0.677)]. When liver steatosis was defined as CAP score ≥274 dB/m, the negative association of serum folate with MAFLD was marginally significant in the fully adjusted model [OR = 0.627 (95% CI: 0.366–1.074)]. In gender-stratified analyses, MAFLD was not associated with serum folate in men. However, folate concentrations were inversely associated with MAFLD in women (Supplementary Table 1). In age-stratified analyses, serum folate was inversely associated with MAFLD in participants < 40 years old, but not in participants ≥ 40 years old (Supplementary Table 2).

**Table 2:** Associations between serum folate levels and MAFLD.

	**Crude**	**Model 1**	**Model 2**
**Serum folate, nmol/L**	**OR (95% CI)**	**OR (95% CI)**	**OR (95% CI)**
**Associations between serum folate level and MAFLD (CAP scores** **≥248 dB/m)**			
T1 (≤ 26.6)	Reference	Reference	Reference
T2 (26.6~43.1)	0.671 (0.498, 0.905)	0.632 (0.434, 0.920)	0.646 (0.429, 0.973)
T3 (> 43.1)	0.726 (0.576, 0.915)	0.508 (0.394, 0.654)	0.521 (0.401, 0.677)
**Associations between serum folate level and MAFLD (CAP scores** **≥274 dB/m)**			
T1 (≤ 26.6)	Reference	Reference	Reference
T2 (26.6~43.1)	0.717 (0.510, 1.008)	0.676 (0.470, 0.973)	0.632 (0.389, 1.026)
T3 (> 43.1)	0.788 (0.547, 1.134)	0.599 (0.382, 0.941)	0.627 (0.366, 1.074)

OR, odds ratio; 95% CI, 95% confidence interval. Model 1 adjusted for: age, sex and race. Model 2 adjusted for: age, sex and race, education, smoking status, and daily alcohol consumption.

The association of serum folate levels with NAFLD was defined as different cutoff values for steatosis (Supplementary Table 3). When liver steatosis was defined as CAP score ≥248 dB/m, T3 group of serum folate had a 38.4% lower risk of NAFLD compared with the T1 group after adjusting for age, sex and race, education, smoking status, and daily alcohol consumption [OR = 0.616 (95% CI: 0.451–0.842)].

### Associations between serum folate levels and MAFLD stratified by vitamin C status

Table 3 showed the effect of vitamin C status on the association between serum folate and MAFLD. In the T1 and T2 groups of vitamin C status, there was no significant association between serum folate and MAFLD in the fully adjusted model, no matter liver steatosis was defined as CAP scores ≥ 248 dB/m or ≥ 274 dB/m. However, in the T3 group of vitamin C status, the T3 group of serum folate had a 63.6% lower risk of MAFLD compared with the T1 group after adjusting for age, sex and race, education, smoking status, and daily alcohol consumption [OR = 0.364 (95% CI: 0.147–.903)], when liver steatosis was defined as CAP score ≥248 dB/m. The result is similar to the above finding when liver steatosis was defined as CAP score ≥ 274 dB/m [OR = 0.209 (95% CI: 0.089–0.491)]. In women, among subjects of T3 group vitamin C status, the T3 group of serum folate was not associated with MAFLD in the fully adjusted model in women when liver steatosis was defined as with CAP scores ≥ 248 dB/m [OR = 0.271 (95% CI: 0.055–1.330)] (Supplementary Table 4). In participants < 40 years old, among subjects of T3 group vitamin C status, the T3 group of serum folate was associated with MAFLD in the fully adjusted model when liver steatosis was defined as with CAP scores ≥ 248 dB/m [OR = 0.283 (95% CI: 0.096–0.837)] (Supplementary Table 5).

**Table 3:** Serum folate in relation to MAFLD according to vitamin C status.

	**Crude**	**Model 1**	**Model 2**
**Serum folate, nmol/L**	**OR (95% CI)**	**OR (95% CI)**	**OR (95% CI)**
**Serum folate in relation to MAFLD according to vitamin C status (CAP scores** **≥248 dB/m)**			
T1 of vitamin C (≤ 39.5 μmol/L)			
T1 (≤ 21.4)	Reference	Reference	Reference
T2 (21.4~31.8)	0.611 (0.317, 1.179)	0.602 (0.313, 1.156)	0.575 (0.274, 1.208)
T3 (>31.8)	0.742 (0.419, 1.313)	0.570 (0.318, 1.020)	0.588 (0.307, 1.128)
T2 of vitamin C (39.5~61.9 μmol/L)
T1 (≤ 28.0)	Reference	Reference	Reference
T2 (28.0~42.0)	0.995 (0.526, 1.881)	0.858 (0.424, 1.734)	0.728 (0.272, 1.949)
T3 (>42.0)	0.977 (0.552, 1.727)	0,671 (0.350, 1.286)	0.489 (0.222, 1.079)
T3 of vitamin C (>61.9 μmol/L)
T1 (≤ 34.0)	Reference	Reference	Reference
T2 (34.0~56.4)	1.045 (0.425, 2.571)	0.729 (0.311, 1.711)	1.264 (0.509, 3.138)
T3 (>56.4)	0.599 (0.316, 1.136)	0.265 (0.117, 0.597)	0.364 (0.147, 0.903)
**Serum folate in relation to MAFLD according to vitamin C status (CAP scores** **≥274 dB/m)**			
T1 of vitamin C (≤ 39.5 μmol/L)			
T1 (≤ 21.4)	Reference	Reference	Reference
T2 (21.4~31.8)	0.602 (0.245, 1.477)	0.578 (0.240, 1.393)	0.561 (0.231, 1.362)
T3 (>31.8)	0.758 (0.376, 1.530)	0.644 (0.308, 1.348)	0.636 (0.296, 1.363)
T2 of vitamin C (39.5~61.9 μmol/L)			
T1 (≤ 28.0)	Reference	Reference	Reference
T2 (28.0~42.0)	0,941 (0.564, 1.570)	0.803 (0.458, 1.409)	0.654 (0.250, 1.710)
T3 (>42.0)	1.099 (0.680, 1.775)	0.766 (0.404, 1.455)	0.670 (0.313, 1.437)
T3 of vitamin C (>61.9 μmol/L)			
T1 (≤ 34.0)	Reference	Reference	Reference
T2 (34.0~56.4)	1.557 (0.578, 4.197)	1.146 (0.441, 2.979)	1.895 (0.772, 4.652)
T3 (>56.4)	0.356 (0.150, 0.845)	0.156 (0.061, 0.398)	0.209 (0.089, 0.491)

OR, odds ratio; 95% CI, 95% confidence interval. Model 1 adjusted for: age, sex and race. Model 2 adjusted for: age, sex and race, education, smoking status, and daily alcohol consumption.

The association between serum folate and NAFLD stratified by vitamin C status was presented in Supplementary Table 6. When liver steatosis was defined as CAP score ≥ 248 dB/m, among subjects of T3 group of vitamin C status, the T3 group of serum folate had a 57.8% lower risk of NAFLD compared with the T1 group in the fully adjusted model [OR = 0.422 (95% CI: 0.223–0.797)]. The association remained unchanged when CAP score ≥274 dB/m was defined as liver steatosis [OR = 0.328 (95% CI: 0.142–0.755)].

The associations between serum vitamin C levels and the prevalence of MAFLD were also investigated (Supplementary Table 7). When liver steatosis was defined as CAP score ≥ 248 dB/m, compared with the serum vitamin C level of T1 group, the prevalence of MAFLD decreased by 50.5% [OR = 0.495 (95% CI: 0.289–0.848)] in the T3 group after adjusting for age, sex and race, education, smoking status, and daily alcohol consumption. The association was marginally significant when liver steatosis was defined as CAP score ≥ 274 dB/m [OR = 0.514 (95% CI: 0.242–1.095)]. In gender-stratified analyses, serum vitamin C was inversely associated with MAFLD in women, but not in men (Supplementary Table 8). In age-stratified analyses, serum vitamin C was inversely associated with MAFLD in participants < 40 years old, but not in participants ≥ 40 years old (Supplementary Table 9).

### Associations with liver fibrosis

There was no association between serum folate and the prevalence of liver fibrosis (Supplementary Table 10). When stratified by serum vitamin C levels, we also did not observe a relationship between serum folate levels and liver fibrosis (Supplementary Table 11). And there was the association between vitamin C and liver fibrosis in T2 group, but not in T3 group (Supplementary Table 12).

## Discussion

As far as we know, it is the first study to investigate the impact of vitamin C status on the association between serum folate and the prevalence of MAFLD, which illustrated that the serum folate level was inversely associated with the risk of developing MAFLD in adults after adjusting for potential confounders. More importantly, when stratified by serum vitamin C levels, the inverse correlation between the serum folate level and MAFLD was only maintained at high serum vitamin C level, but not at low serum vitamin C levels. Based on the interesting phenomenon, we speculate that the protective effect of increased serum folate on MAFLD may be largely dependent on serum vitamin C levels.

To our knowledge, no epidemiologic study with a large number of samples has investigated the association between the serum folate level and MAFLD, and the results of previous studies are contradictory. A single-center cross-sectional study with 30 NAFLD patients and 24 healthy controls matched for sex, age, BMI, and waist circumference showed serum folate levels were not significantly different between the two groups (16). However, serum folate levels were lower in obese patients with severe NAFLD compared with subjects with normal liver or mild liver alterations (15). A study of Chinese adults (70 subjects undergoing liver biopsy, and 130 subjects undergoing proton magnetic resonance spectroscopy (^1^H-MRS) for liver fat measurement) demonstrated that serum folic acid levels were inversely correlated with the degree of hepatic steatosis (29). In addition, the addition of serum folic acid to the NAFLD prediction score significantly improved diagnostic performance (29). Similar to these two studies, we concluded that serum folate levels were inversely associated with the prevalence of MAFLD in populations with a relatively large number of samples. Animal studies also support these findings. As reported, chronic folate deficiency led to liver steatosis in mice (30). Moreover, folic acid supplementation reduced liver lipid accumulation and liver inflammation induced by a high-fat diet in mice (31). An 8-week short-term animal study showed that folic acid supplementation significantly reduced steatosis and improved liver function in the rats fed with a high-fructose diet (32).

Reportedly, the occurrence and development of NAFLD are related to epigenetic mechanisms, one of which is DNA methylation (33). Hypermethylation and hypomethylation are associated with gene silencing and gene activation, respectively (34, 35). Folate is considered to be a methyl donor for DNA methylation, and can affect the expression of cellular genes, resulting in a series of effects. The underlying mechanism may be that folate plays an important role in the synthesis of purine and thymidylate and in DNA methylation (36), and folate deficiency can lead to high expression of genes related to hepatic fat synthesis, resulting in hepatic steatosis (37). In addition, folate deficiency reduces the level of phosphatidy-lethanolamine N-methyltransferase, thereby reducing the methylation of phosphatidylethanolamine to phosphatidylcholine, which ultimately affects low-density lipoprotein secretion and causes liver fat accumulation (38, 39). Folic acid supplementation can regulate the transcription of NADPH oxidase, effectively reduce the oxidative stress caused by high-fat diet (40), increase AMP levels, and activate LKB1 to restore AMPK activation in the liver, thereby improving cholesterol and glucose metabolism, which may slow down the pathogenesis of NAFLD (41).

As an antioxidant, vitamin C reduces oxidative stress and prevents chronic inflammatory diseases (42). The relationship between vitamin C and liver steatosis has been investigated. An observational study with 72 NAFLD patients indicated that the prevalence of serum vitamin C deficiency and insufficient vitamin C intake was very high (43). Multifactorial regression analysis demonstrated vitamin C intake was negatively associated with the prevalence of NAFLD in 797 subjects ≥18 years old (44). In another cross-sectional study of 4,494 subjects, the increased serum vitamin C concentrations were negatively association with MAFLD among adults in the United States (19), which is consistent to our results. More interestingly, we found that the inverse correlation between the serum folate concentration and MAFLD was maintained only in subjects with higher serum vitamin C status, but not in subjects with low serum vitamin C levels. Therefore, sufficient serum vitamin C may be an important prerequisite for the protective effect of serum folate on MAFLD. It implies that the simultaneous supplementation of VC and folic acid in multivitamin tablets may be reasonable. Our results may provide new ideas for clinical nutritional treatment of MAFLD.

In gender-stratified analyses, although serum folate and vitamin C were inversely associated with MAFLD in women, the inverse correlation between the serum folate level and MAFLD was not maintained at high serum vitamin C level when stratified by serum vitamin C levels. We speculate that this may be caused by the small number of participants after stratification. In age-stratified analyses, the inverse correlation between the serum folate level and MAFLD was maintained at high serum vitamin C level when stratified by serum vitamin C levels.

There are several studies on the association of folate and liver fibrosis. A study showed that folate supplementation can increase the level of fusion protein Syntaxin 17 in the liver, which is important in reversing the development of liver inflammation and fibrosis (45). However, folate levels did not differ significantly in fibrosis stages in a study of 30 patients with biopsy-proven NAFLD and 24 healthy controls (16). The results of this study are consistent with our result, but we need further research.

The association between vitamin C and liver fibrosis has also been investigated. Forty-five NASH patients participated in the 6-month trial, with 23 receiving vitamin E (1000IU) and vitamin C (1,000 mg), and 22 receiving placebo. The fibrosis scores significantly improved in the vitamin group (46). However, a previous cross-sectional study of 789 people confirmed that dietary vitamin C intake was not associated with liver fibrosis (47). When stratified by serum vitamin C levels, we did not observe the association between serum folate levels and liver fibrosis. It is suggested that supplementing vitamin C and folate at the same time has no effect on liver fibrosis.

There are several strengths of this research. Firstly, it is the first study to investigate the impact of serum vitamin C status on the association of serum folate levels with the prevalence of MAFLD. Secondly, the recently proposed definition MAFLD was adopted, which further assures the novelty of our research. Thirdly, NHANES is representative of the civilian and non-institutionalized national population, which makes our results more representative and persuasive. However, there are some limitations. First, it is a cross-sectional study, so no causal relationship between serum folate levels or serum vitamin C levels and MAFLD can be established. Second, although we tried our best to adjust for the potential confounders, not all of the confounding factors can be included. Third, data such as drug therapy, lifestyle and dietary habit were not available, which may have impacted our findings.

In conclusion, serum folate level is inversely associated with the prevalence of MAFLD, especially in participants with sufficient serum vitamin C. It may be necessary to maintain sufficient serum vitamin C for ensuring the protective effect of increased folate on MALFD. However, more studies are needed to validate this.

## Data availability statement

The datasets presented in this study can be found in online repositories (https://www.cdc.gov/nchs/nhanes/index.htm).

## Ethics statement

The studies involving human participants were reviewed and approved by National Center for Health Statistics. The patients/participants provided their written informed consent to participate in this study. Written informed consent was obtained from the individual(s), and minor(s)' legal guardian/next of kin, for the publication of any potentially identifiable images or data included in this article.

## Author contributions

YJ, HC, XC, GY, CS, HD, and FT participated in the data collection and analysis. XC and GY validated the data. YJ and HC drafted the manuscript. HW and JS revised the manuscript and served as scientific advisors. All authors contributed to the study conception and design.

## Funding

This work was supported by the National Natural Science Foundation of China (82170800), Guangdong Basic and Applied Basic Research Foundation (2021A1515110682), and Research Initiation Project of Shunde Hospital of Southern Medical University (SRSP2021001).

## Conflict of interest

The authors declare that the research was conducted in the absence of any commercial or financial relationships that could be construed as a potential conflict of interest.

## Publisher's note

All claims expressed in this article are solely those of the authors and do not necessarily represent those of their affiliated organizations, or those of the publisher, the editors and the reviewers. Any product that may be evaluated in this article, or claim that may be made by its manufacturer, is not guaranteed or endorsed by the publisher.

## Abbreviations

MAFLD: Metabolic dysfunction-associated fatty liver disease
NHANES: National Health and Nutrition Examination Survey
NAFLD: Non-alcoholic fatty liver disease
NCHS: National Center for Health Statistics
MEC: Mobile Examination Center
VCTE: vibration-controlled transient elastography
CAP: controlled attenuation parameter
LSM: liver stiffness measurements
TC: total cholesterol
TG: triglyceride
HDL-C: high-density lipoproteins cholesterol
LDL-C: low-density lipoproteins cholesterol
BMI: body mass index.
