# Supplementary material for: Associations of serum folate and vitamin C levels with metabolic dysfunction-associated fatty liver disease in US adults: A nationwide cross-sectional study
Source: Front Public Health. 2022 Oct 26;10:1022928. doi: 10.3389/fpubh.2022.1022928 (PMC9643688; doi:10.3389/fpubh.2022.1022928)
Supplement: Supplementary file 1 [file Table_1.docx]

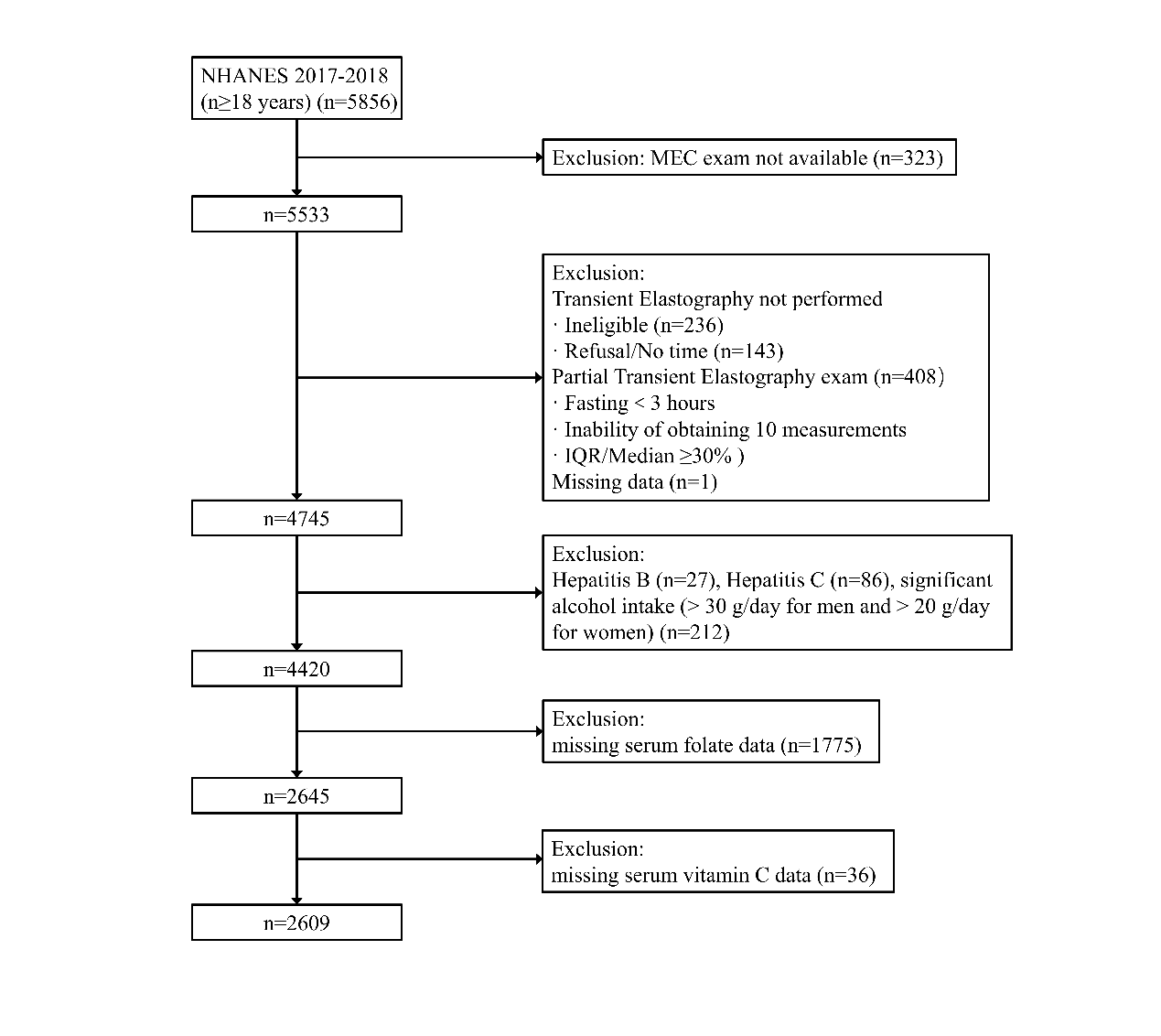


**Supplementary Fig. 1** Flow-chart of the NAFLD participants

**Supplementary Table 1.** Associations between serum folate levels and MAFLD stratified by gender.

|  | **Crude** | **Model 1** | **Model 2** |
| --- | --- | --- | --- |
| **Serum folate, nmol/L** | **OR (95%CI)** | **OR (95% CI)** | **OR (95% CI)** |
| **Associations between serum folate level and MAFLD (CAP scores ≥ 248 dB/m)**  **men** | | | |
| T1 (≤ 25.9) | Reference | Reference | Reference |
| T2 (25.9~40.8) | 0.994 (0.581, 1.534) | 0.916 (0.544, 1.543) | 0.871 (0.425, 1.783) |
| T3 (> 40.8) | 1.164 (0.592, 2.289) | 0.704 (0.324, 1.53) | 0.609 (0.245, 1.515) |
| **women** | | | |
| T1 (≤27.0) | Reference | Reference | Reference |
| T2 (27.0~45.3) | 0.507 (0.327, 0.788) | 0.466 (0.280, 0.774) | 0.486 (0.271, 0.870) |
| T3 (> 45.3) | 0.551 (0.382, 0.793) | 0.412 (0.292, 0.582) | 0.535 (0.362, 0.791) |

OR, odds ratio; 95% CI, 95% confidence interval. Model 1 adjusted for: age and race. Model 2 adjusted for: age and race, education, smoking status, and daily alcohol consumption.

**Supplementary Table 2.** Associations between serum folate levels and MAFLD stratified by age.

|  | **Crude** | **Model 1** | **Model 2** |
| --- | --- | --- | --- |
| **Serum folate, nmol/L** | **OR (95%CI)** | **OR (95% CI)** | **OR (95% CI)** |
| **Associations between serum folate level and MAFLD (CAP scores ≥ 248 dB/m)**  **Age < 40 years** | | | |
| T1 (≤ 25.2) | Reference | Reference | Reference |
| T2 (25.2~37.7) | 0.607 (0.374, 0.984) | 0.572 (0.342, 0.957) | 0.531 (0.297, 0.951) |
| T3 (> 37.7) | 0.409 (0.290, 0.578) | 0.396 (0.269, 0.585) | 0.338 (0.227, 0.505) |
| **Age ≥ 40 years** | | | |
| T1 (≤28.2) | Reference | Reference | Reference |
| T2 (28.2~47.6) | 0.910 (0.637, 1.301) | 0.899 (0.624, 1.294) | 1.021 (0.570, 1.829) |
| T3 (> 47.6) | 0.685 (0.400, 1.173) | 0.689 (0.398, 1.191) | 0.759 (0.406, 1.420) |

OR, odds ratio; 95% CI, 95% confidence interval. Model 1 adjusted for: gender and race. Model 2 adjusted for: gender and race, education, smoking status, and daily alcohol consumption.

**Supplementary Table 3.** Associations between serum folate levels and NAFLD

|  | **Crude** | **Model 1** | **Model 2** |
| --- | --- | --- | --- |
| **Serum folate, nmol/L** | **OR (95%CI)** | **OR (95% CI)** | **OR (95% CI)** |
| **Associations between serum folate level and NAFLD (CAP scores ≥ 248 dB/m)** | | | |
| T1 (≤26.8) | Reference | Reference | Reference |
| T2 (26.8~43.5) | 0.768 (0.521, 1.130) | 0.717 (0.446, 1.152) | 0.779 (0.471, 1.288) |
| T3 (> 43.5) | 0.799 (0.658, 0.971) | 0.578 (0.433, 0.770) | 0.616 (0.451, 0.842) |
| **Associations between serum folate level and NAFLD (CAP scores ≥ 274 dB/m)** | | | |
| T1 (≤26.8) | Reference | Reference | Reference |
| T2 (26.8~43.5) | 0.702 (0.504, 0.978) | 0.649 (0.463, 0.909) | 0.626 (0.408, 0.960) |
| T3 (> 43.5) | 0.846 (0.603, 1.187) | 0.659 (0.428, 1.015) | 0.711 (0.414, 1.220) |

OR, odds ratio; 95% CI, 95% confidence interval. Model 1 adjusted for: age, sex and race. Model 2 adjusted for: age, sex and race, education, smoking status, and daily alcohol consumption.

**Supplementary Table 4.** Serum folate in relation to MAFLD according to vitamin C status in women

|  | **Crude** | **Model 1** | **Model 2** |
| --- | --- | --- | --- |
| **Serum folate, nmol/L** | **OR (95%CI)** | **OR (95% CI)** | **OR (95% CI)** |
| **Serum folate in relation to MAFLD according to vitamin C status (CAP scores ≥ 248 dB/m)** | | | |
| T1 of vitamin C (≤42.6μmol/L) |  |  |  |
| T1 (≤20.8) | Reference | Reference | Reference |
| T2 (20.8~31.2) | 0.606 (0.298, 1.231) | 0.586 (0.287, 1.197) | 0.515 (0.265, 1.001) |
| T3 (>31.2) | 0.619 (0.240, 1.598) | 0.479 (0.177, 1.298) | 0.567 (0.264, 1.218) |
| T2 of vitamin C (42.6~65.3μmol/L) |  |  |  |
| T1 (≤29.0) | Reference | Reference | Reference |
| T2 (29.0~46.3) | 0.869 (0.380, 1.985) | 0.795 (0.337, 1.874) | 0.536 (0.212, 1.354) |
| T3 (>46.3) | 1.162 (0.404, 3.343) | 0.939 (0.319, 2.765) | 0.902 (0.291, 2.793) |
| T3 of vitamin C (>65.3μmol/L) |  |  |  |
| T1 (≤35.0) | Reference | Reference | Reference |
| T2 (35.0~57.7) | 0.480 (0.119, 1.929) | 0.407 (0.102 ,1.620) | 0.804 (0.130, 4.969) |
| T3 (>57.7) | 0.439 (0.180, 1.073) | 0.219 (0.067, 0.712) | 0.271 (0.055, 1.330) |

OR, odds ratio; 95% CI, 95% confidence interval. Model 1 adjusted for: age and race. Model 2 adjusted for: age and race, education, smoking status, and daily alcohol consumption.

**Supplementary Table 5.** Serum folate in relation to MAFLD according to vitamin C status in participants < 40 years old

|  | **Crude** | **Model 1** | **Model 2** |
| --- | --- | --- | --- |
| **Serum folate, nmol/L** | **OR (95%CI)** | **OR (95% CI)** | **OR (95% CI)** |
| **Serum folate in relation to MAFLD according to vitamin C status (CAP scores ≥ 248 dB/m)** | | | |
| T1 of vitamin C (≤41.3μmol/L) |  |  |  |
| T1 (≤20.4) | Reference | Reference | Reference |
| T2 (20.4~29.5) | 0.661 (0.221, 1.974) | 0.671 (0.221, 2.035) | 0.730 (0.182, 2.923) |
| T3 (>29.5) | 0.560 (0.196, 1.599) | 0.538 (0.183, 1.587) | 0.538 (0.146, 1.978) |
| T2 of vitamin C (41.3~60.8μmol/L) |  |  |  |
| T1 (≤26.7) | Reference | Reference | Reference |
| T2 (26.7~37.4) | 0.801 (0.361, 1.775) | 0.761 (0.369, 1.569) | 0.362 (0.122, 1.072) |
| T3 (>37.4) | 1.015 (0.579, 1.781) | 1.072 (0.587, 1.958) | 0.803 (0.386, 1.669) |
| T3 of vitamin C (>60.8μmol/L) |  |  |  |
| T1 (≤30.2) | Reference | Reference | Reference |
| T2 (30.2~46.0) | 1.138 (0.330, 3.923) | 1.078 (0.281, 4.135) | 0.918 (0.148, 5.693) |
| T3 (>46.0) | 0.430 (0.128, 1.445) | 0.408 (0.114, 1.451) | 0.283 (0.096, 0.837) |

OR, odds ratio; 95% CI, 95% confidence interval. Model 1 adjusted for: gender and race. Model 2 adjusted for: gender and race, education, smoking status, and daily alcohol consumption.

**Supplementary Table 6.** Serum folate in relation to NAFLD according to vitamin C status

|  | **Crude** | **Model 1** | **Model 2** |
| --- | --- | --- | --- |
| **Serum folate, nmol/L** | **OR (95%CI)** | **OR (95% CI)** | **OR (95% CI)** |
| **Serum folate in relation to NAFLD according to vitamin C status (CAP scores ≥ 248 dB/m)** | | | |
| T1 of vitamin C (≤40.40μmol/L) |  |  |  |
| T1 (≤21.6) | Reference | Reference | Reference |
| T2 (21.6~31.8) | 0.777 (0.416, 1.453) | 0.786 (0.418, 1.478) | 0.710 (0.375, 1.343) |
| T3 (>31.8) | 0.801 (0.451, 1.424) | 0.650 (0.361, 1.172) | 0.718 (0.417, 1.235) |
| T2 of vitamin C (40.40~62.5μmol/L) |  |  |  |
| T1 (≤28.7) | Reference | Reference | Reference |
| T2 (28.7~42.7) | 0.855 (0.428, 1.708) | 0.743 (0.377, 1.465) | 0.717 (0.304, 1.689) |
| T3 (>42.7) | 0.944 (0.577, 1.543) | 0.685 (0.388, 1.208) | 0.549 (0.276, 1.094) |
| T3 of vitamin C (>62.5μmol/L) |  |  |  |
| T1 (≤34.2) | Reference | Reference | Reference |
| T2 (34.2~56.5) | 0.997 (0.377, 2.634) | 0.649 (0.220, 1.912) | 1.236 (0.402, 3.805) |
| T3 (>56.5) | 0.619 (0.257, 1.487) | 0.295 (0.139, 0.627) | 0.422 (0.223, 0.797) |
| **Serum folate in relation to NAFLD according to vitamin C status (CAP scores ≥ 274 dB/m)** | | | |
| T1 of vitamin C (≤40.40μmol/L) |  |  |  |
| T1 (≤21.6) | Reference | Reference | Reference |
| T2 (21.6~31.8) | 0.696 (0.284, 1.707) | 0.690 (0.283, 1.683) | 0.653 (0.281, 1.521) |
| T3 (>31.8) | 0.771 (0.363, 1.637) | 0.676 (0.314, 1.455) | 0.675 (0.312, 1.460) |
| T2 of vitamin C (40.40~62.5μmol/L) |  |  |  |
| T1 (≤28.7) | Reference | Reference | Reference |
| T2 (28.7~42.7) | 0.692 (0.364, 1.315) | 0.570 (0.318, 1.021) | 0.443 (0.189,1.036) |
| T3 (>42.7) | 0.957 (0.597, 1.534) | 0.684 (0.369, 1.268) | 0.614 (0.290,1.300) |
| T3 of vitamin C (>62.5μmol/L) |  |  |  |
| T1 (≤34.2) | Reference | Reference | Reference |
| T2 (34.2~56.5) | 1.723 (0.711, 4.175) | 1.296 (0.517, 3.244) | 2,636 (1.076, 6.459) |
| T3 (>56.5) | 0.522 (0.199, 1.368) | 0.253 (0.092, 0.698) | 0.328 (0.142, 0.755) |

OR, odds ratio; 95% CI, 95% confidence interval. Model 1 adjusted for: age, sex and race. Model 2 adjusted for: age, sex and race, education, smoking status, and daily alcohol consumption.

**Supplementary Table 7.** Associations between serum vitamin C levels and MAFLD

|  | **Crude** | **Model 1** | **Model 2** |
| --- | --- | --- | --- |
| **vitamin C, μmol/L** | **OR (95%CI)** | **OR (95% CI)** | **OR (95% CI)** |
| **Associations between serum vitamin C level and MAFLD (CAP scores ≥ 248 dB/m)** | | | |
| T1 (≤39.5) | Reference | Reference | Reference |
| T2 (39.5~61.9) | 0.818 (0.520, 1.288) | 0.845 (0.539, 1.325) | 0.818 (0.496, 1.349) |
| T3 (> 61.9) | 0.576 (0.363, 0.914) | 0.517 (0.328, 0.814) | 0.495 (0.289, 0.848) |
| **Associations between serum vitamin C level and MAFLD (CAP scores ≥ 274 dB/m)** | | | |
| T1 (≤39.5) | Reference | Reference | Reference |
| T2 (39.5~61.9) | 0.721 (0.462, 1.125) | 0.728 (0.455, 1.164) | 0.684 (0.396, 1.183) |
| T3 (> 61.9) | 0.614 (0.339, 1.112) | 0.568 (0.319, 1.010) | 0.514 (0.242, 1.095) |

OR, odds ratio; 95% CI, 95% confidence interval. Model 1 adjusted for: age, sex and race. Model 2 adjusted for: age, sex and race, education, smoking status, and daily alcohol consumption.

**Supplementary Table 8.** Associations between serum vitamin C levels and MAFLD stratified by gender.

|  | **Crude** | **Model 1** | **Model 2** |
| --- | --- | --- | --- |
| **vitamin C, μmol/L** | **OR (95%CI)** | **OR (95% CI)** | **OR (95% CI)** |
| **Associations between serum vitamin C level and MAFLD (CAP scores ≥ 248 dB/m)**  **men** | | | |
| T1 (≤34.4) | Reference | Reference | Reference |
| T2 (34.4~55.5) | 1.417 (0.723, 2.777) | 1.510 (0.705, 3.239) | 1.604 (0.737, 3.490) |
| T3 (> 55.5) | 1.003 (0.495, 2.032) | 0.872 (0.442, 1.718) | 0.929 (0.400, 2.157) |
| **women** | | | |
| T1 (≤42.6) | Reference | Reference | Reference |
| T2 (42.6~65.3) | 0.505 (0.295, 0.865) | 0.531 (0.316, 0.891) | 0.507 (0.281, 0.915) |
| T3 (> 65.3) | 0.397 (0.216, 0.729) | 0.344 (0.191, 0.619) | 0.300 (0.165, 0.546) |

OR, odds ratio; 95% CI, 95% confidence interval. Model 1 adjusted for: age and race. Model 2 adjusted for: age and race, education, smoking status, and daily alcohol consumption.

**Supplementary Table 9.** Associations between serum vitamin C levels and MAFLD stratified by age.

|  | **Crude** | **Model 1** | **Model 2** |
| --- | --- | --- | --- |
| **vitamin C, μmol/L** | **OR (95%CI)** | **OR (95% CI)** | **OR (95% CI)** |
| **Associations between serum vitamin C level and MAFLD (CAP scores ≥ 248 dB/m)**  **Age < 40 years** | | | |
| T1 (≤41.3) | Reference | Reference | Reference |
| T2 (41.3~60.8) | 0.700 (0.451, 1.085) | 0.701 (0.430, 1.142) | 0.663 (0.352, 1.251) |
| T3 (> 60.8) | 0.322 (0.197, 0.527) | 0.318 (0.191, 0.529) | 0.281 (0.141, 0.558) |
| **Age ≥ 40 years** | | | |
| T1 (≤37.6) | Reference | Reference | Reference |
| T2 (37.6~62.5) | 1.018 (0.504, 2.058) | 1.010 (0.500, 2.043) | 0.988 (0.486, 2.010) |
| T3 (> 62.5) | 0.724 (0.348, 1.507) | 0.729 (0.346, 1.536) | 0.696 (0.321, 1.507) |

OR, odds ratio; 95% CI, 95% confidence interval. Model 1 adjusted for: gender and race. Model 2 adjusted for: gender and race, education, smoking status, and daily alcohol consumption.

**Supplementary Table 10.** Associations between serum folate levels and liver fibrosis

|  | **Crude** | **Model 1** | **Model 2** |
| --- | --- | --- | --- |
| **Serum folate, nmol/L** | **OR (95%CI)** | **OR (95% CI)** | **OR (95% CI)** |
| **Associations between serum folate level and liver fibrosis (LSM ≥ 7 KPa)** | | | |
| T1 (≤26.6) | Reference | Reference | Reference |
| T2 (26.6~43.1) | 0.794 (0.468, 1.348) | 0.764 (0.427, 1.366) | 0.810 (0.455, 1.444) |
| T3 (> 43.1) | 0.749 (0.469, 1.197) | 0.607 (0.386, 0.957) | 0.687 (0.435, 1.085) |
| **Associations between serum folate level and liver fibrosis (LSM ≥ 8** **KPa)** | | | |
| T1 (≤26.6) | Reference | Reference | Reference |
| T2 (26.6~43.1) | 1.287 (0.475, 3.489) | 1.249 (0.442, 3.533) | 1.076 (0.417, 2.777) |
| T3 (> 43.1) | 1.062 (0.501, 2.249) | 0.899 (0.439, 1.841) | 0.854 (0.362, 2.017) |

OR, odds ratio; 95% CI, 95% confidence interval. Model 1 adjusted for: age, sex and race. Model 2 adjusted for: age, sex and race, education, smoking status, and daily alcohol consumption.

**Supplementary Table 11.** Serum folate in relation to liver fibrosis according to vitamin C status

|  | **Crude** | **Model 1** | **Model 2** |
| --- | --- | --- | --- |
| **Serum folate, nmol/L** | **OR (95%CI)** | **OR (95% CI)** | **OR (95% CI)** |
| **Serum folate in relation to liver fibrosis according to vitamin C status (LSM ≥ 7** **KPa)** | | | |
| T1 of vitamin C (≤39.5μmol/L) |  |  |  |
| T1 (≤21.4) | Reference | Reference | Reference |
| T2 (21.4~31.8) | 1.273 (0.631, 2.568) | 1.251 (0.641, 2.443) | 1.581 (0.817, 3.061) |
| T3 (>31.8) | 1.335 (0.569, 3.131) | 1.153 (0.532, 2.502) | 1.107 (0.510, 2.399) |
| T2 of vitamin C (39.5~61.9μmol/L) |  |  |  |
| T1 (≤28.0) | Reference | Reference | Reference |
| T2 (28.0~42.0) | 1.009 (0.436, 2.332) | 0.910 (0.371, 2.233) | 0.843 (0.296, 2.402) |
| T3 (>42.0) | 1.160 (0.554, 2.427) | 0.890 (0.436, 1.817) | 0.792 (0.354, 1.773) |
| T3 of vitamin C (>61.9μmol/L) |  |  |  |
| T1 (≤34.0) | Reference | Reference | Reference |
| T2 (34.0~56.4) | 0.886 (0.276, 2.849) | 0.721 (0.175, 2.973) | 0.911 (0.303, 2.743) |
| T3 (>56.4) | 0.753 (0.116, 4.896) | 0.684 (0.083, 5.646) | 1.155 (0.267, 5.009) |
| **Serum folate in relation to liver fibrosis according to vitamin C status (LSM ≥ 8** **KPa)** | | | |
| T1 of vitamin C (≤39.5μmol/L) |  |  |  |
| T1 (≤21.4) | Reference | Reference | Reference |
| T2 (21.4~31.8) | 0.880 (0.334, 2.319) | 0.847 (0.33, 2.174) | 0.991 (0.384, 2.562) |
| T3 (>31.8) | 1.456 (0.527, 4.022) | 1.179 (0.459, 3.031) | 0.764 (0.287, 2.032) |
| T2 of vitamin C (39.5~61.9μmol/L) |  |  |  |
| T1 (≤28.0) | Reference | Reference | Reference |
| T2 (28.0~42.0) | 1.117 (0.407, 3.061) | 1.013 (0.351, 2.929) | 0.873 (0.246, 3.096) |
| T3 (>42.0) | 1.293 (0.484, 3.455) | 1.001 (0.372, 2.695) | 0.845 (0.210, 3.400) |
| T3 of vitamin C (>61.9μmol/L) |  |  |  |
| T1 (≤34.0) | Reference | Reference | Reference |
| T2 (34.0~56.4) | 0.932 (0.217, 4.004) | 0.938 (0.216, 4.066) | 0.579 (0.108, 3.094) |
| T3 (>56.4) | 0.983 (0.154, 6.295) | 1.499 (0.213, 10.542) | 1.078 (0.242, 4.808) |

OR, odds ratio; 95% CI, 95% confidence interval. Model 1 adjusted for: age, sex and race. Model 2 adjusted for: age, sex and race, education, smoking status, and daily alcohol consumption.

**Supplementary Table 12.** Associations between serum vitamin C levels and liver fibrosis

|  | **Crude** | **Model 1** | **Model 2** |
| --- | --- | --- | --- |
| **vitamin C, μmol/L** | **OR (95%CI)** | **OR (95% CI)** | **OR (95% CI)** |
| **Associations between serum vitamin C level and liver fibrosis (LSM ≥ 7** **KPa)** | | | |
| T1 (≤39.5) | Reference | Reference | Reference |
| T2 (39.5~61.9) | 0.362 (0.226, 0.579) | 0.362 (0.220, 0.596) | 0.343 (0.195, 0.602) |
| T3 (> 61.9) | 0.703 (0.35, 1.409) | 0.677 (0.332, 1.377) | 0.636 (0.266, 1.522) |
| **Associations between serum vitamin C level and liver fibrosis (LSM ≥ 8** **KPa)** | | | |
| T1 (≤39.5) | Reference | Reference | Reference |
| T2 (39.5~61.9) | 0.415 (0.264, 0.651) | 0.415 (0.261, 0.659) | 0.524 (0.265, 1.035) |
| T3 (> 61.9) | 0.821 (0.497, 1.359) | 0.800 (0.465, 1.376) | 1.074 (0.415, 2.780) |

OR, odds ratio; 95% CI, 95% confidence interval. Model 1 adjusted for: age, sex and race. Model 2 adjusted for: age, sex and race, education, smoking status, and daily alcohol consumption.
